# Natural Phenylethanoid Supplementation Alleviates Metabolic Syndrome in Female Mice Induced by High-Fructose Diet

**DOI:** 10.3389/fphar.2022.850777

**Published:** 2022-07-19

**Authors:** Xiujun Zhan, Mingshuai He, Jierong Pei, Wenjing Fan, Charity Ngina Mwangi, Peng Zhang, Xin Chai, Miaomiao Jiang

**Affiliations:** ^1^ State Key Laboratory of Component-Based Chinese Medicine, Tianjin University of Traditional Chinese Medicine, Tianjin, China; ^2^ Haihe Laboratory of Modern Chinese Medicine, Tianjin, China

**Keywords:** salidroside, fructose, gut microbiota, metabolic syndrome, hepatic protection

## Abstract

Tyrosol (T), hydroxytyrosol (H), and salidroside (S) are typical phenylethanoids and also powerful dietary antioxidants. This study aimed at evaluating the influence of three natural phenylethanoids, which are dietary phenylethanoids of natural origins, on reversing gut dysbiosis and attenuating nonalcoholic fatty liver features of the liver induced by metabolic syndrome (MetS) mice. C57BL/6J female mice induced with high-fructose diet were established and administrated with salidroside, tyrosol, and hydroxytyrosol for 12 weeks, respectively. Biochemical analysis showed that S, T, and H significantly improved glucose metabolism and lipid metabolism, including reduced levels of total cholesterol insulin (INS), uric acid, low-density lipoprotein cholesterol (LDL-C), and aspartate aminotransferase (ALT). Histopathological observation of the liver confirmed the protective effects of S, T, and H against hepatic steatosis, which were demonstrated by the results of metabolomic analysis, such as the improvement in glycolysis, purine metabolism, bile acid, fatty acid metabolism, and choline metabolism. Additionally, 16S rRNA gene sequence data revealed that S, T, and H could enhance the diversity of gut microbiota. These findings suggested that S, T, and H probably suppress lipid accumulation and have hepatoprotective effects and improve intestinal microflora disorders to attenuate metabolic syndromes.

## Introduction

Metabolic syndrome (MetS) is a group of metabolic risk factors, including obesity, insulin resistance, dyslipidemia, hypertension, and hyperuricemia (Huang, 2009). The prevalence of MetS is growing at an alarming rate worldwide in recent years, and it has now become a truly global problem (Saklayen, 2018). Research has shown that high consumption of large amounts of saturated fat and carbohydrates has been considered the main predisposing factor to MetS (Hannou et al., 2018). Fructose is closely related to people’s lives. It not only exists in some vegetables, fruits, and honey but is also widely added to processed foods and beverages. Supplementing some beneficial dietary ingredients to ameliorate the effects of fructose may be an effective strategy for the prevention of MetS (Pan and Kong, 2018).

Tyrosol (T), hydroxytyrosol (H), and salidroside (S) are not only typical phenylethanoids but also one of the strongest dietary antioxidants. They are found in plants such as olive, green tea, *Ligustrum lucidum* W.T.Aiton [Oleaceae], *Rhodiola crenulata* (Hook.f. & Thomson) H. Ohba [Crassulaceae], and both red and white wines. Studies have confirmed that they display a range of pharmacological properties, including anti-inflammatory, anti-tumor, anti-hypoxia, hepatoprotective, and antioxidant activities (Kalaiselvan et al., 2016; Chen et al., 2019; Markovic et al., 2019; Bertelli et al., 2020; Yu et al., 2020; Zhang et al., 2020; Zhang et al., 2021; Li et al., 2022). These have been attributed to increased attention to the interest in dietary tyrosol, hydroxytyrosol, or salidroside. The European Food Safety Authority (EFSA) has greatly emphasized the effect of tyrosol and its derivates, such as the effect of hydroxytyrosol to protect low-density lipoproteins (LDLs) from oxidation and pointed out that the dietary ingestion of 5 mg is enough to obtain the benefits (Efsa, 2011). Hydroxytyrosol has an ortho-diphenolic structure and high antioxidant activity (Balducci et al., 2018). Analogously, hydroxytyrosol, extracted from olive leaves and oil, has been proved to prevent MetS onset by preserving the glycemic index, reducing triglyceride (TG) levels, and preventing LDL oxidation and inflammation (Jemai et al., 2008). Salidroside, a glucoside of tyrosol, is one of the main active ingredients of the medicinal herb, *Rhodiola crenulata* (Hook. f. & Thomson) H. Ohba (Chen et al., 2008). Moreover, salidroside exhibits various biological activities and is used for the prophylaxis and therapeutics of many diseases, including non-alcoholic fatty liver disease (NAFLD), type 2 diabetes, and cardiovascular diseases (Zheng et al., 2018; Bai et al., 2019; Pu et al., 2020).

It is well established that the liver is the primary site of dietary fructose metabolism. Interestingly, a recent study using isotope tracing and mass spectrometry found that most dietary fructose is metabolized by the small intestine (Jang et al., 2018). Only when the intake of fructose overwhelms intestinal fructose absorption and clearance, the excess fructose spills over to the liver and microbiota (Ferraris et al., 2018). Pharmacokinetics and bioavailability showed that when evaluating the bioavailability of tyrosol, hydroxytyrosol, or salidroside, the efficiency of intestinal absorption is not the only factor to be considered. We also need to take into account the enterohepatic circulation (Miro-Casas et al., 2003; Aleman-Jimenez et al., 2021). Therefore, in our study, we paid more attention to tyrosol, hydroxytyrosol, and salidroside influences on intestinal–hepatic metabolism. We provided salidroside, hydroxytyrosol, and tyrosol to female mice and fed an HFru diet to determine whether salidroside, hydroxytyrosol, or tyrosol intervention could recover liver injury and modulate the gut microbiota in HFru diet-induced female mice.

## Materials and Methods

### Chemicals and Materials

In total, 60 female C57BL/6J mice (6 weeks, 18–19 g) were purchased from Beijing Vital River Laboratory Animal Technology Co., Ltd. (Beijing, China). The normal diet (ND) was purchased from Xiao Shu Youtai (Beijing, China) Biological Technology Co., Ltd., and the high-fructose diet (HFru) was obtained from Tianjin Aoyide Experimental Products Co., Ltd. (Tianjin, China).

Tyrosol (purity ≥98%, S31415) and pioglitazone (purity≥98%, B21435) were purchased from Shanghai Yuanye Biological Technology Co., Ltd. (Shanghai, China). Salidroside (purity ≥98%) and hydroxytyrosol (purity ≥98%) were obtained from Sichuan Victory Biological Technology Co., Ltd. (Sichuan, China). Deuterium oxide (D_2_O, 99.9% atom %D) and 3-(trimethylsilyl)-propionic-2,2,3,3-d_4_ acid sodium salt (TSP-d_4_, 98% atom %D) were purchased from Cambridge Isotope Laboratories (Cambridge, FL, United States). Methanol was purchased from Sigma-Aldrich (St. Louis, MO, United States).

### Dietary Intervention Study of Mice

All the animals were maintained at a controlled temperature (23 ± 2°C) with a 12 h light/dark period and acclimated with *ad libitum* access to a standard chow diet and water for 1 week. They were randomly and equally divided into six groups, including the control group (fed with ND diet; i.g. homologous saline per day), model group (fed with HFru diet; i.g. homologous saline per day), positive group (fed with HFru diet; i.g. 6.00 mg/kg dosage of pioglitazone), salidroside group (fed with HFru diet; i.g. 50 mg/kg dosage of salidroside), tyrosol group (fed with HFru diet; i.g. 23 mg/kg dosage of tyrosol), and hydroxytyrosol group (fed with HFru diet; i.g. 26 mg/kg dosage of hydroxytyrosol). The feeding cycle lasted for 8 weeks. The dose of salidroside was determined according to the previous animal experimental results of the research group (Song et al., 2021). Tyrosol and hydroxytyrosol were calculated based on the molar conversion with salidroside. The compositions of HFru were fructose (60%), casein (20.7%), cellulose (8%), lard (5%), minerals (0.5%), vitamins (0.1%), DL-methionine (0.03%), and food coloring. *All animal procedures and testing were performed according to the Laboratory Animals Center at the Institute of Radiation Medicine Chinese Academy of Medical Sciences, Tianjin, China.*

The body weight was measured every week. At the end of treatment, the animals were kept in an empty cage without bedding to gather fresh stool samples into sterile tubes, and they were snap-frozen in liquid nitrogen. All animals were fasted overnight before being euthanized. The tail cutting method was used to determine the level of fasted blood glucose. Blood was collected by plucking the eyeballs and then centrifuged to yield serum samples. Liver samples, dissected from the thoracic and abdominal cavities, were immersed immediately in liquid nitrogen and stored at −80°C for further analysis. *The study was approved by the Animal Ethics Committee of the Institute of Radiation Medicine Chinese Academy of Medical Sciences (Tianjin, China, Approval No: IRM-DWLL-2020127).*

### Serum and Liver Biochemistry

The obtained mouse blood was centrifuged at 4000 rpm for 10 min, and the supernatant was collected. The serum levels of total cholesterol (TC), triglyceride (TG), low-density lipoprotein-cholesterol (LDL-C), high-density lipoprotein-cholesterol (HDL-C), alanine aminotransferase (ALT), and glucose (GLU) of the mice were determined by commercial kits on a biochemical automatic analyzer (Hitachi, Japan, 7020). The levels of serum uric acid (UA) and insulin (INS) were measured in strict accordance with the kit instructions.

The liver tissue levels of alanine aminotransferase (ALT), xanthine oxidase (XOD), tumor necrosis factor-α (TNF-α), total bile acid (TBA), farnesoid X receptor (FXR), and monooxygenase-3 (FMO3) were determined in accordance with kit instructions.

### Hepatic Histopathological Examination

Fresh liver samples were fixed in 10% para formaldehyde, embedded in paraffin, sectioned into 5 μm thickness, and stained with hematoxylin/eosin (H&E). The sliced sections were observed using a Nikon Ci-L microscope (Japan) and analyzed using the HMIAS-2000 high-resolution digital microscope image analysis system.

### Hepatic Metabolomic Profiling

The 100 mg liver tissue was homogenized by 800 μL of pre-cold CH_3_OH/H_2_O (2:1) on ice. The extractive solution was vortexed for 30 s and centrifuged at 12,000 r and 4°C for 10 min. The supernatant of 800 μL in a new tube was dried under nitrogen, and 600 μL of phosphate-buffered saline (pH 7.4) containing 0.01% sodium 3-trimethysilyl [2,2,3,3-d4] propionate (TSP-d4) was added. The solution was vortexed and centrifuged again. The amount of 500 μL supernatant was pipetted and transferred into a 5-mm NMR tube. The NMR tube was stored at 4°C for the test.

^1^H-NMR spectra were recorded at 300 K on a Bruker Avance III 600 MHz spectrometer, operating at 600.13 MHz for proton, equipped with a cryogenic probe (Bruker, Biospin, Germany). A CPMG (Carr–Purcell–Meiboom–Gill) pulse train was adopted and scanned 64 times (NS) with a 90° pulse width (P1) of 14.09 μs, acquisition time (AQ) of 3.41 s, a relaxation delay time of (D1) of 5 s, spectral width (SWH) of 9615 Hz, and receiving gain of 188 and a sampling data point of 65,536.

The NMR original data were processed by MestReNova 12.0.1 software (Mestrelab S.L., Spain). The process was carried out by phase correction and baseline correction before the methyl resonance of TSP-d4 was referenced to δ 0.00 ppm. The 1H NMR spectra from δ 0.7–9.0 ppm were bucketed into bins with an integral step of 0.001 ppm. The normalized integrity data were imported into SIMCA (version 14.1, Sweden) to perform partial least squares discriminant analysis (PLS-DA). Differential metabolites were screened by variable importance in the project (VIP) > 1 and *t*-test (*p* < 0.05). An FDR-adjusted *p*-value, which was obtained by a *t*-test performed in MetaboAnalyst 5.0, was used in the study. In addition, the determination of trimethylamine N-oxide (TMAO) and betaine in liver samples referred to a reported article (He et al., 2021).

### Bioinformatic Gut Microbiota Analyses

Total DNA for gut microbiota analysis was extracted from approximately 100 mg of feces. To generate representative complete sequences, the raw tags were improved and managed using FLASH software (Version 1.2.11, United States). We accepted the Quantitative Insights into Microbial Ecology (QIIME) (Version 1.7.0) quality control process to obtain clean tags. Subsequently, the clean tags, in comparison with the reference database using the UCHIME algorithm (UCHIME Algorithm), were used to remove the chimera sequences to obtain effective tags. The effective tags were determined quantitatively and analyzed using the Quanti-Fluorimeter and the Hiseq2500 system (Illumina, Inc., San Diego, CA, United States). Sequences with ≥97% similarity were assigned to the same operational taxonomic units (OTUs). QIIME software displayed was used to calculate the alpha diversity index, including Chao1, Shannon, Simpson, ACE, and beta diversity index (principal component analysis (PCA)) and non-metric multidimensional scaling (NMDS). The linear discriminant analysis (LDA) and LDA effect size (LEfSe) methods were applied to analyze the metagenomic biomarker among groups (Galaxy Online Analysis Platform, http://huttenhower.sph.harvard.edu/galaxy/), and the selected differences were sorted by linear discriminant analysis (LDA) > 4.0.

### Statistical Analysis

The obtained data were analyzed by GraphPad Prism 9.0 (San Diego, CA, United States) and expressed as mean ± SD. One-way analysis of variance (ANOVA) with the Dunnett test was employed to evaluate the significance of differences among animal groups.

Differences were considered statistically significant at *p* < 0.05. Partial least squares discriminant analysis (PLS-DA) was performed on SIMCA 14.1 (Sweden).

A value of *P or ^#^ *p* < 0.05, **P or ^##^ *p* < 0.01, and ***P or ^###^ *p* < 0.001 was considered a statistically significant difference for all analyses.

## Results

### Effect on Body Weight, Fasting Blood Glucose, and Serum Biochemical Indicators in Mice

MetS is a combination of a series of clinical risk factors, including elevated blood pressure, obesity, high TG, and high blood glucose levels. Tables 1, 2; Supplementary Figure S1A indicated that the control group mice had the lowest ALT, TC, TG, LDL-C, HDL-C, UA, GLU, FBG, and INS serum levels, and the model group mice had the highest ALT, TC, TG, LDL-C, HDL-C, UA, GLU, FBG, and INS levels (*p* < 0.05). After the administration of salidroside, tyrosol, or hydroxytyrosol, the levels of ALT, TG, LDL-C, UA, GLU, FBG, and INS in metabolic syndrome mice significantly decreased (*p* < 0.05), while the level of TC only showed a downward trend. It was worth noting that the change in the HDL-C level was different from that reported in the literature. Although pioglitazone or different extracts had no obvious effect on the improvement of body weight, body weight had a down tendency in the medicated administration group, especially in the salidroside group. In a comprehensive comparison, the effect of the salidroside group was stronger than that of the tyrosol or hydroxytyrosol group.

**TABLE 1:** Body weight, fasting blood glucose, and serum metabolic parameters in mice.

Group	C	M	P	S	T	H
FBG (mmol/L)	7.15 ± 1.57	9.84 ± 2.04**	6.85 ± 2.00^##^	6.46 ± 1.64^###^	6.05 ± 1.35^###^	6.36 ± 1.42^###^
Final weight (g)	21.20 ± 1.08	22.18 ± 1.29	21.20 ± 0.80	22.36 ± 1.40	21.31 ± 1.23	20.95 ± 1.69
TG (mmol/L)	0.94 ± 0.24	1.27 ± 0.31*	0.93 ± 0.15^##^	0.82 ± 0.23^###^	0.69 ± 0.16^###^	0.78 ± 0.20^###^
TC (mmol/L)	2.57 ± 0.41	3.56 ± 0.49*	3.19 ± 0.44	3.32 ± 0.39	3.49 ± 0.34	3.39 ± 0.25
HDL-C (mmol/L)	1.14 ± 0.16	1.48 ± 0.19***	1.19 ± 0.16^##^	1.27 ± 0.17^#^	1.29 ± 0.12	1.27 ± 0.12^#^
LDL-C (mmol/L)	0.24 ± 0.02	0.41 ± 0.07***	0.25 ± 0.05^###^	0.32 ± 0.04^##^	0.33 ± 0.05^#^	0.32 ± 0.08^#^
ALT (U/L)	36.50 ± 7.11	47.67 ± 8.53*	34.10 ± 8.63^###^	31.67 ± 3.50^###^	37.22 ± 5.52^#^	38.22 ± 8.44^#^
GLU (mmol/L)	4.81 ± 0.75	6.67 ± 1.41*	4.56 ± 0.97^##^	3.53 ± 1.47^###^	4.29 ± 1.67^##^	4.23 ± 1.32^##^
UA (mmol/L)	106.40 ± 14.26	145.20 ± 18.52***	111.90 ± 15.40^###^	107.40 ± 22.78^###^	112.60 ± 14.58^###^	111.00 ± 13.85^###^
INS (mIU/L)	1.82 ± 0.65	5.12 ± 0.57***	1.99 ± 0.76^###^	2.60 ± 0.65^###^	1.44 ± 0.28^###^	1.48 ± 0.68^###^

Values presented are the mean ± standard deviation (N ≥ 8/group). *p < 0.05, **p < 0.01, ***p < 0.001, vs. C; ^#^ *p* < 0.05, ^##^ *p* < 0.01, ^###^ *p* < 0.001 vs. M. P: mice treated with pioglitazone (6.00 mg/kg); S: mice treated with salidroside (50 mg/kg); T: mice treated with tyrosol (23 mg/kg); H: mice treated with hydroxytyrosol (26 mg/kg). FBG: fasting blood glucose; ALT: alanine aminotransferase; TC: total cholesterol; TG: triglyceride; HDL-C: high-density lipoprotein cholesterol; LDL-C: low-density lipoprotein cholesterol; GLU: glucose; UA: uric acid; INS: insulin.

**TABLE 2:** Effect of three phenylethanoids on hepatic metabolic parameters in mice.

Groups	C	M	P	S	T	H
ALT (U/L)	26.20 ± 5.33	33.30 ± 3.20**	27.70 ± 4.47^#^	27.80 ± 4.52^#^	27.90 ± 4.28^#^	28.20 ± 4.02^#^
XOD (U)	17.10 ± 4.48	24.30 ± 2.58***	17.90 ± 4.18^##^	18.10 ± 4.01^##^	18.00 ± 3.50^##^	18.50 ± 3.50^##^
TNF-α (pg/ml)	878.90 ± 38.19	1318.00 ± 83.51***	777.60 ± 141.70^###^	773.70 ± 99.79^###^	790.40 ± 157.70^###^	847.60 ± 125.20^###^
TBA (μmol/L)	0.16 ± 0.05	0.15 ± 0.04***	0.16 ± 0.05^##^	0.16 ± 0.05^##^	0.16 ± 0.05^##^	0.16 ± 0.05^#^
FXR (ng/ml)	5.26 ± 0.44	4.18 ± 0.74*	5.32 ± 0.29^#^	5.48 ± 0.86^#^	5.30 ± 1.14^#^	5.24 ± 0.90^#^
FMO3 (U)	17.88 ± 1.88	20.83 ± 1.84*	16.53 ± 2.64^###^	13.32 ± 1.37^###^	14.57 ± 2.46^###^	13.63 ± 1.64^###^

Values presented are the mean ± standard deviation (N ≥ 8/group). *p < 0.05, **p < 0.01, ***p < 0.001, vs. C; ^#^ *p* < 0.05, ^##^ *p* < 0.01, ^###^ *p* < 0.001 vs. M. P: mice treated with pioglitazone (6.00 mg/kg); S: mice treated with salidroside (50 mg/kg); T: mice treated with tyrosol (23 mg/kg); H: mice treated with hydroxytyrosol (26 mg/kg). ALT: alanine aminotransferase; XOD: xanthine oxidase; TNF-α: tumor necrosis factor-α; TBA: total bile acid; FXR: farnesoid X receptor; FMO3: flavin monooxygenase 3.

### Effect on Hepatic Biochemical Indicators

Table 1; Supplementary Figure S1B depicted that the levels of ALT, XOD, TNF-α, and FMO_3_ in the serum of healthy mice were the lowest, while TBA and FXR levels were the highest. Mice in the model group showed a contrary trend. The serum levels of ALT, XOD, TNF-α, and FMO_3_ were the highest, and the TBA and FXR levels were the lowest in the model group. After the administration of salidroside, tyrosol, and hydroxytyrosol to the respective groups, the levels of ALT, XOD, TNF-α, and FMO_3_ in metabolic syndrome mice markedly decreased, while the levels of TBA and FXR significantly increased (*p* < 0.05). The salidroside group had the strongest action to improve these indicators, which was significantly stronger than the tyrosol or hydroxytyrosol group.

### Effect on Liver Histopathology

**FIGURE 1:**
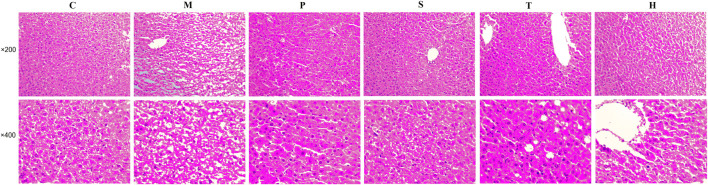
After treatment with salidroside, tyrosol, and hydroxytyrosol for 12 weeks, liver tissues were stained with H&E staining (×200 and ×400). C: control; M: model; P: pioglitazone; S: salidroside; T: tyrosol; H: hydroxytyrosol.

The liver pathological sections of mice in each group were observed under a 200x and 400x high power microscope (Figure 1). In normal chow-fed mice, hepatic cords were radially arranged around a central vein. Also, the portal canal area had no inflammatory cell infiltration. However, the accumulation of round and tense vacuoles in the hepatocyte cytoplasm could be observed in the liver tissue of model mice, and the lipid droplets increased obviously, and the inflammatory cells infiltrated significantly. These undesirable conditions were attenuated by salidroside, tyrosol, or hydroxytyrosol treatment, especially the salidroside group that had stronger inhibitory effects on liver lesions and regulated the MetS better than the tyrosol and hydroxytyrosol groups.

### Metabonomic Changes in Liver and Differential Metabolite Analysis

The changes of metabolites in the liver were determined to assess the effect of salidroside, tyrosol, and hydroxytyrosol. Supplementary Figure S2; Supplementary Table S1 showed the signal attribution of typical ^1^H-NMR spectra of liver tissue extracts of six groups of mice. According to the literature, public databases (The Human Metabolome Database, HMDB), and the two-dimensional spectrum, a total of 52 metabolites were identified, mainly including amino acids, organic acids, alkaloids, sugars, and nucleotides. To investigate the differences in metabolic profiling between different groups, the PLS-DA model and permutation test were performed and are shown in Supplementary Figure S3; Supplementary Table S2x. VIP >1 and *p* < 0.05 were chosen as screening criteria to screen the differential metabolites (Supplementary Tables S3–7). A total of 29 differential metabolites were identified between the control group and model group, which were 3-hydroxybutyrate, leucine, lactate, threonine, alanine, proline, *O*-acetylcarnitine, succinate, trimethylamine, glutathione, glutamine, creatine, creatinine, choline, phosphorylcholine, glycerophosphocholine (GPC), taurine, trimethylamine-*N*-oxide (TMAO), glycerol, glycine, *N*-phosphocreatine, threonate, inosine, glucose, serine, anserine, xanthine, oxypurinol, and niacinamide. It was observed that HFru mice clearly distinguished from chow-fed mice, and we suggested that certain metabolic disorders had occurred in the model group.

**FIGURE 2:**
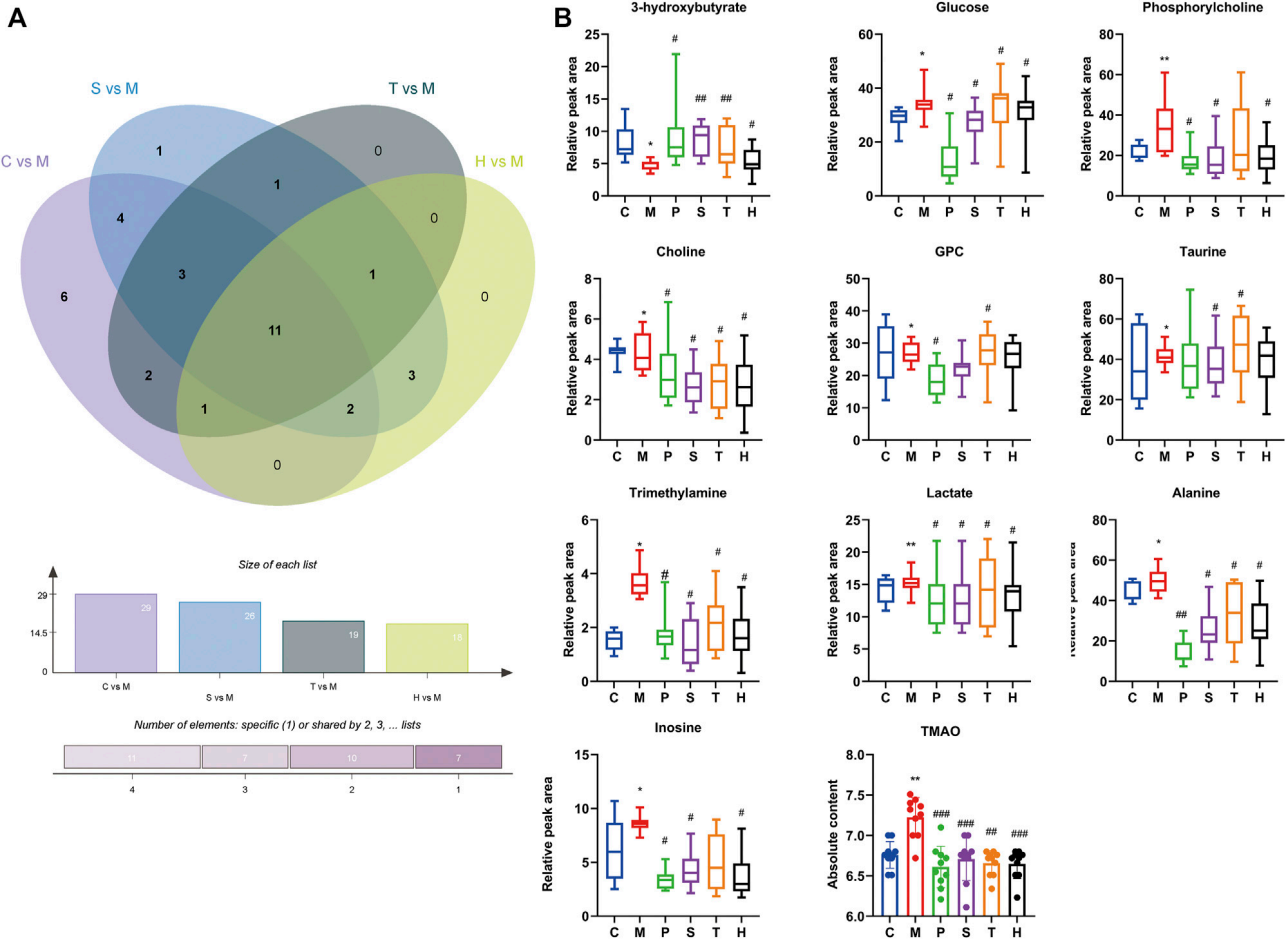
Differential metabolites in mice liver between different groups. **(A)** Intersection differential metabolites changes in C vs. M, S vs. M, T vs. M, and H vs. M by Venny analysis. **(B)** Individual relative content changes of differential metabolites in common were represented by the boxplot. *p < 0.05, **p < 0.01, ***p < 0.001, vs. C; #*p* < 0.05, ##*p* < 0.01, ###*p* < 0.001 vs. M. C: control; M: model; P: pioglitazone; S: salidroside; T: tyrosol; H: hydroxytyrosol.

There were 11 shared metabolites between C vs*.* M, S vs*.* M, T vs*.* M, and H vs*.* M found by Venny analysis (http://www.ehbio.com/test/venn/#/), including 3-hydroxybutyrate, lactate, alanine, trimethylamine, choline, phosphorylcholine, GPC, taurine, TMAO, inosine, and glucose (Figure 2). Also, the changes of other differential metabolites are shown in Supplementary Figure S4. By the intervention of salidroside, tyrosol, and hydroxytyrosol, 26, 19, and 18 differential metabolites could be regulated back, respectively. The results suggested that the three natural phenylethanoids could significantly suppress the increased hepatic absolute content of TMAO (*p* < 0.01, *p* < 0.001), while only the salidroside group caused a significant reduction in betaine absolute content (*p* < 0.01). The results showed that the salidroside group had better improving metabolism abnormalities than the tyrosol and hydroxytyrosol groups.

**FIGURE 3:**
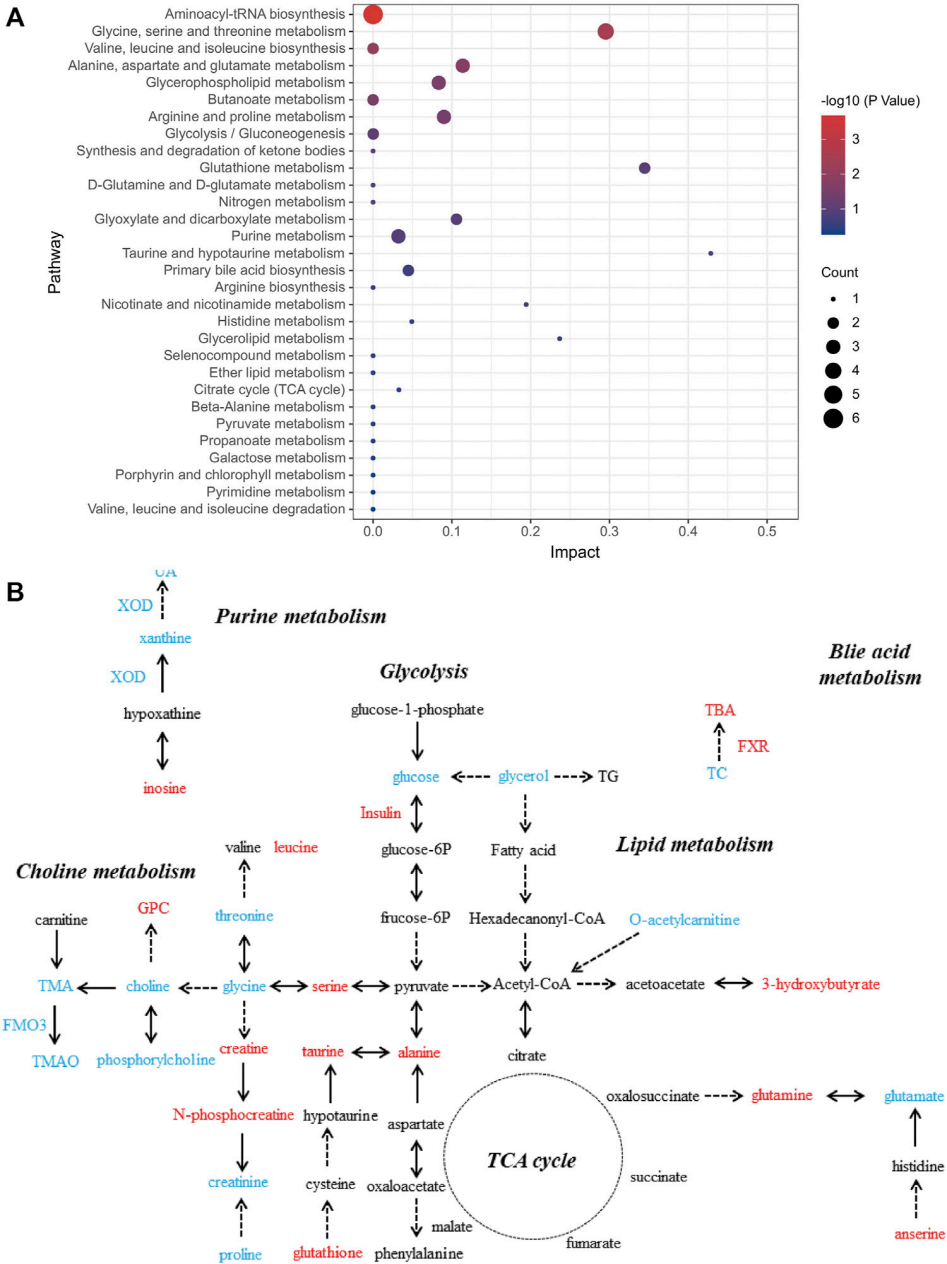
Analysis of related metabolic pathways. **(A)** Pathway analysis for group C vs. M by using the KEGG database. The horizontal axis represents impact; a higher impact indicates a greater degree of enrichment. The point size represents the number of differentially expressed metabolites in KEGG, and the colors of the points correspond to different *p*-value ranges. **(B)** Which metabolites have caused change after treatment with salidroside, tyrosol, and hydroxytyrosol for 12 weeks is shown. The red font represents the upregulation of differential metabolites or biochemical indexes after administration, while the blue font represents the reverse.

We further investigated the role of these differential metabolites in metabolic pathways using the pathway analysis module of MetaboAnalyst 5.0. Figure 3 shows that the protective effect of salidroside, tyrosol or hydroxytyrosol on the mice model of MetS may be attributed to the regulation of these differential metabolites and their related metabolic pathways, including bile acid metabolism, choline metabolism, glycolysis, purine metabolism, amino acid metabolism, lipid metabolism, taurine metabolism, and the tricarboxylic acid cycle.

### Impact of Salidroside, Tyrosol, or Hydroxytyrosol Intervention on the Gut Microbial Community

**FIGURE 4:**
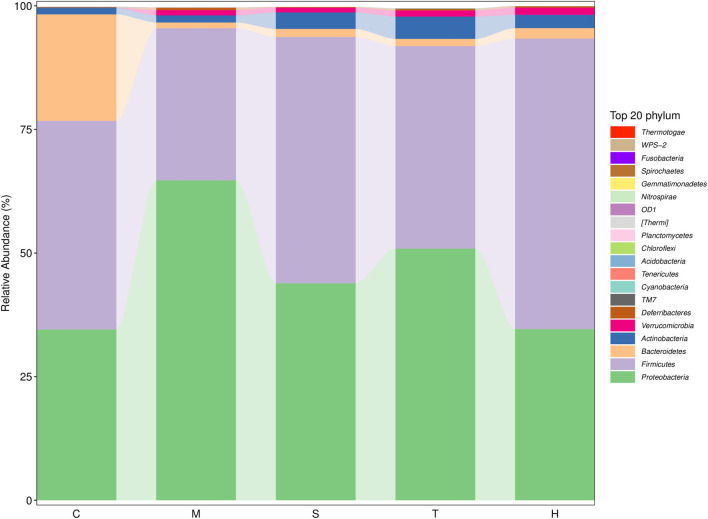
Histogram of mean value of intestinal microflora enrichment in mice at the phylum level in each group. C: control; M: model; S: salidroside; T: tyrosol; H: hydroxytyrosol.

**FIGURE 5:**
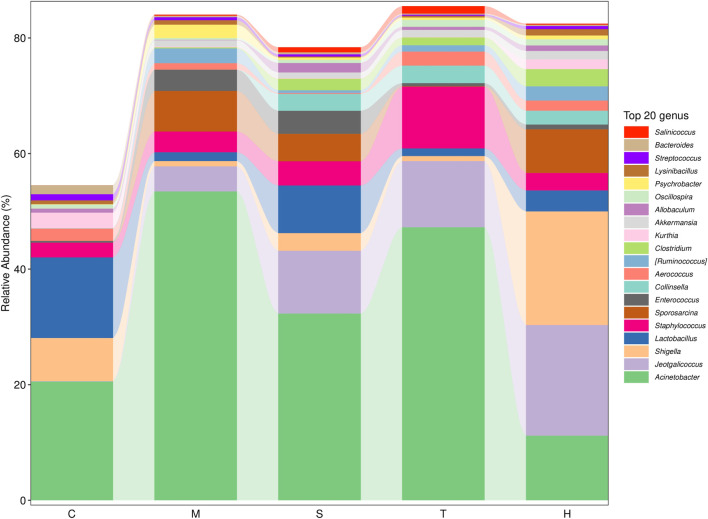
Histogram of mean value of intestinal microflora enrichment in mice at the genus level in each group. C: control; M: model; S: salidroside; T: tyrosol; H: hydroxytyrosol.

Gut microbiota are strictly associated with a fructose diet. To analyze the effect of salidroside, tyrosol or hydroxytyrosol on the composition of gut microbiota, an analysis of gut microbiota 16S rRNA in HFru-mice feces was performed. Shannon and Simpson’s index revealed that the fecal microbiota diversity in the model group was lower than that in the control group (Supplementary Figure S5). In addition, Chao 1 and observed species index indicated that salidroside, tyrosol, or hydroxytyrosol could affect community richness (Supplementary Figure S5), especially from the alpha diversity index, the hydroxytyrosol group changed the most, and there was a significant difference. PCoA analysis showed that HFru intervention results had significant cluster separation from those of the control group (Supplementary Figure S6A). These conditions were partially recovered by salidroside, tyrosol, or hydroxytyrosol treatment, suggesting that salidroside, tyrosol, or hydroxytyrosol could alter the gut microbiota compositions to some extent. The NMDS plot of the microbial compositions of the 30 samples revealed that salidroside, tyrosol, or hydroxytyrosol treatment groups and the control groups demonstrated different microbiome compositions (Supplementary Figure S6B). The Venn diagram showed that a total of 204 OTUs were shared by all groups (Supplementary Figure S6C). In addition, the main phyla observed in our study include *Firmicutes*, *Bacteroidetes*, *Proteobacteria*, *Actinobacteria*, and *Verrucomicrobia* (Figure 4). The largest proportions of the phylum *Amoebacteria* and *Proteobacteria* were found in each group. Particularly, a high-fructose diet could significantly decrease the relative abundance of *Firmicutes* and *Bacteroidetes* while significantly increasing the relative abundance of *Proteobacteria*. At the genus level (Figure 5), we found that the most abundant bacteria were mainly *Acinetobacter*, *Jeotgalicoccus*, *Lactobacillus*, *Staphylococcus*, and *Sporosarcina*. Detailed analysis at phylum and genus levels revealed that salidroside, tyrosol, and hydroxytyrosol groups had their relative regulation of bacteria. Salidroside and hydroxytyrosol treatment had an advantage over upregulating the abundance of *Lactobacillus*, and tyrosol and hydroxytyrosol groups downregulated the abundance of *Enterococcus*. The intervention of tyrosol could enhance the abundance of *Staphylococcus* while decreasing the abundance of *Sporosarcina*. Also, the hydroxytyrosol group increased the abundance of *Shigella*. It was noted that treatment groups could upregulate the abundance of *Jeotgalicoccus* and downregulate the abundance of *Acinetobacter*. Next, LEfSe analysis and cladogram analysis were performed to identify fecal microbial taxa that accounted for the greatest differences among all the groups. Our results indicated that there were 14, 16, 7, 16, and 15 significant differences in the control, high-fructose diet, salidroside−50 mg/kg, tyrosol−23 mg/kg, and hydroxytyrosol−26 mg/kg groups, respectively (Supplementary Figures 7, 8). In general, salidroside, tyrosol, or hydroxytyrosol intervention influenced the shaping of gut microbiota.

**FIGURE 6:**
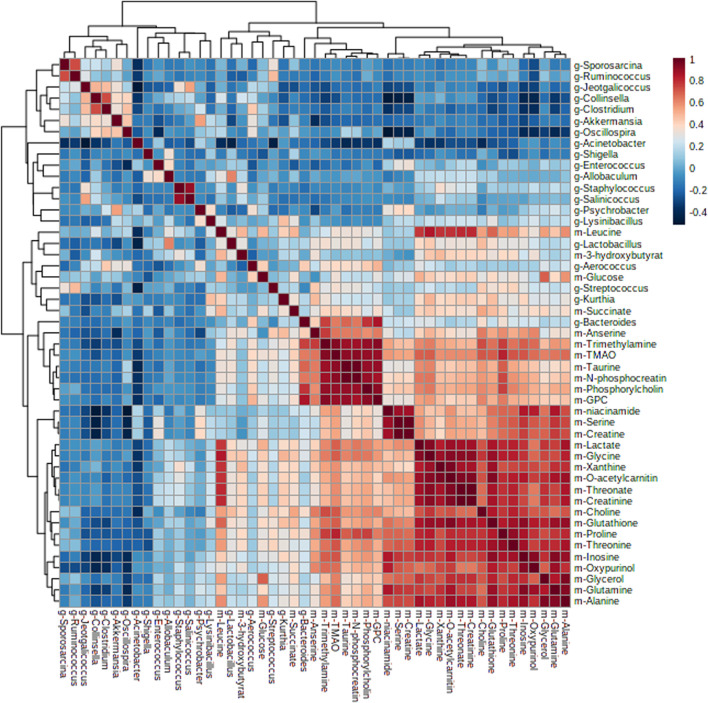
Heatmap of correlation analysis results between intestinal flora and liver metabolites.

Furthermore, by Pearson correlation analysis, we found that there was a potential link between changes in the intestinal flora and liver metabolites. As depicted in Figure 6, there was a negative correlation between *Acinetobacter* and taurine; *Oscillospira* and niacinamide; *Collinsella* and niacinamide, serine, creatine, inosine, oxypurinol, glutamine, trimethylamine, and TMAO; *Fusobacterium* and glutathione, niacinamide, serine, creatine, inosine, oxypurinol, glutamine, trimethylamine, TMAO, proline, and threonine; *Sporosarcina* and oxypurinol; *Ruminococcus* and GPC, niacinamide, serine, inosine, oxypurinol; and *Akkermansia* and inosine, oxypurinol, trimethylamine, and anserine, while *Lactobacillus* was positively correlated with GPC and *Kurthia* was positively correlated with inosine and anserine. These metabolites were mainly involved in amino acid tRNA biosynthesis, glycine, serine, and threonine metabolism, arginine and proline metabolism, and glyoxylate and dicarboxylate metabolism.

## Discussion

Fructose would seem a safe and natural sweetener in daily life, but mounting evidence suggests that high fructose intake has an unfavorable impact on metabolism and a strong correlation with various diseases (Lim et al., 2010; Madero et al., 2012; Mirtschink et al., 2018), especially with MetS. Previous studies have shown that tyrosol, hydroxytyrosol, and salidroside could alleviate the features of MetS such as dyslipidemia, hyperglycemia, insulin resistance, or adiposity (Chandramohan et al., 2015; Ju et al., 2017; Peyrol et al., 2017). However, rodent experiments with pure tyrosol, hydroxytyrosol, and salidroside to elucidate the roles between the liver intestine and its gut microbiota is lacking. Therefore, a well-characterized MetS model was established to be explored. In addition, male and female mice were screened. Biochemical indices and pathological sections (Supplementary Figure S9) showed that female mice were more likely to succeed in modeling.

High fructose consumption could promote fat accumulation, particularly in the liver, and induce hepatic pathological changes. In addition, hepatic fructolysis, in contrast to the metabolism of glucose, is not only more rapid but also bypasses insulin control (Hannou et al., 2018). More importantly, its breakdown metabolites could stimulate a high burden of lipogenesis and uric acid production (Rutledge and Adeli, 2007), and this process cannot be restricted. Obtained results showed that the model groups had high levels of FBG, GLU, TC, TG, LDL-C, and UA in serum, and this can serve as a reminder of hyperglycemia, hyperlipidemia, and hyperuricemia. In particular, we observed increased XOD activity in the liver which is closely related to UA. These symptoms play an indispensable role in the occurrence and development of MetS. In other words, long-term high-fructose feeding could disturb the body’s lipid metabolism and glycometabolism balance. It was noted that the level of HDL-C significantly increased, and this was contrary to our expectation. The specific reasons for this phenomenon are also unclear. The body weight of model groups only displayed a tendency to gain weight. The reason why the body weight did not increase significantly may be the reduction of food intake in the later period. The change in food intake during the whole experiment was not taken into account in this study, which was one of our limitations. Interestingly, recent research demonstrated that HFru-fed C57BL/6J mice had higher blood glucose, endotoxin levels, fat mass, dyslipidemia, and glucose intolerance without changes in body weight, which may be associated with gut microbiota according to experimental data (Do et al., 2018). After 84 days of diet intervention, tyrosol, hydroxytyrosol, or salidroside could suppress the elevation of UA, TG, and LDL-C levels to maintain the body’s lipid metabolism steady state and reduce GLU, FBG, and INS levels. Over-production of UA directly inhibits insulin signaling in hepatocytes, so UA is referred to as a risk factor for insulin resistance. Insulin resistance is also a key pathological event in MetS. However, the content of TC was not improved through drug intervention. As reported, excessive TG deposition can contribute to accelerating the formation of a fatty liver (Vergani, 2019), which is consistent with the HE staining of our liver tissue. Based on these observations, we found that salidroside showed a better protective effect than tyrosol and hydroxytyrosol.

Metabolomics is considered an important tool to study alterations in biochemical pathways intrinsic to the pathophysiology of MetS (Stechemesser et al., 2017; Capel et al., 2020). So, based on the high repeatability as well as the non-destructive and non-invasive nature of the NMR spectrum, our study used NMR metabolomics to evaluate changes in liver metabolites. The experimental outcomes suggested that tyrosol, hydroxytyrosol, and salidroside may be effective in regulating the fructose-induced metabolic disorder to normal levels. Further analysis based on differential metabolites suggested that long-term HFru intake could trigger glucose metabolism and lipid metabolism disorders and have a negative effect on amino acid metabolism. The alterations in the amino acid profile may play a crucial part in participating in the development and progression of MetS (Ntzouvani et al., 2017; Roberts et al., 2020). Therefore, some amino acids could be considered potential markers for MetS risk. In the present study, salidroside, tyrosol, and hydroxytyrosol groups significantly downregulated the relative concentration of glycine, serine, glutamine, alanine, and serine that are strongly associated with dyslipidemia by inhibiting some enzymes in the liver that are activated due to excessive HFru intake. These results are consistent with a large cross-sectional study (Tai et al., 2010). The detailed mechanism of how amino acid metabolism and its products contribute to disease needs to be investigated further. Moreover, it was reported that TMAO had a positive correlation with the etiology of cardiovascular and other diseases (Ufnal et al., 2015). The level of TMAO is influenced by a variety of factors including liver flavin monooxygenase activity, food, and gut microbial flora (Janeiro et al., 2018). In our study, salidroside, tyrosol, and hydroxytyrosol intervention significantly decreased the expression of FMO_3_ in the liver and prevented mice from the accumulation of hepatic TMAO in large amounts. It is worth noting that these differential metabolites are more or less linked to the gut microbiota. A study that used 16S rDNA amplicon sequencing and metabolomic profiling revealed that a kind of purified extract could alleviate gut dysbiosis and regulate metabolism to ameliorate MetS (Zeng et al., 2020). Therefore, the gut microbiota may become a promising target in drug therapy to improve MetS. It is important to recognize that the gut microbiome is a complex and susceptive ecosystem, especially it is easily affected by dietary properties (Zmora et al., 2019). Although salidroside, tyrosol, and hydroxytyrosol possess poor absorption and low bioavailability, we thought they have potential influence on the remodeling of gut microbiota.

In humans and rodents, five bacterial phyla predominate in the gut: *Firmicutes*, *Bacteroidetes*, *Actinobacteria*, *Proteobacteria*, and *Verrucomicrobia*. *Firmicutes* (mainly Gram-positive) and *Bacteroidetes* (Gram-negative) are the predominant phyla in the feces of the host. A growing body of evidence makes it clear that an increase in the *Firmicutes*-to-*Bacteroidetes* ratio was confirmed to be linked with MetS in mice experiments (Turnbaugh et al., 2008; Trompette et al., 2014) and human trials (Mariat et al., 2009; Vaiserman et al., 2020). While using 16S rRNA sequencing to compare gut microbial compositions, we found that salidroside-, tyrosol-, and hydroxytyrosol-treated groups did not decrease the *Firmicutes*-to-*Bacteroidetes* ratio. The reasons for this are still unclear. Treatment with salidroside, tyrosol, and hydroxytyrosol reduced *Proteobacteria* abundance, which was reported to take an active part in glucose homeostasis. The increasing trend of gut *Proteobacteria* reflects an energy disequilibrium in the body and an unstable microbial community. Hence, salidroside, tyrosol, and hydroxytyrosol intervention could alleviate the energy imbalance caused by the high-fructose diet. At the genus level, *Acinetobacter* and *Lactobacillus* were the crucial genuses responding to salidroside and hydroxytyrosol treatment, respectively. Our findings showed that salidroside and hydroxytyrosol could exert metabolic protection by lowering pathogenic bacteria or elevating the beneficial ones in gut microbiota. However, tyrosol had little effect on the beneficial intestinal bacteria as well as pathogenic bacteria. The data from our study revealed that a small group difference in phytochemicals could make a big influence on gut microbiota, especially the phenolic hydroxyl group. Therefore, when assessing the therapeutic action of phytochemicals, it is of great importance to take the role of gut microbiota into account. It has also been proposed that tyrosol is the active ingredient of salidroside, and the two are converted into each other in the body. However, the effect of tyrosol on gut microbiota was far less than that of salidroside. This fact has indicated that the metabolism of salidroside *in vivo* is complex and variable, and this may be related to the dose added to the diet. Additionally, hydroxytyrosol was found to be non-genotoxic and non-mutagenic when its concentrations exceeded those attainable after dietary intake (Auñon-Calles et al., 2013). This may be one of the reasons why the regulatory effect of hydroxytyrosol on intestinal flora is evident. Using Pearson correlation analysis, we found there was a potential link between changes in the intestinal flora and liver metabolites. For example, it was well-known that phosphatidylcholine is metabolized by intestinal microbes, and the resulting metabolites are choline and betaine. Meanwhile, choline and betaine are also metabolized by gut microflora into trimethylamine before it is converted to trimethylamine N-oxide (TMAO) by hepatic flavin monooxygenase. In the model group, phosphatidylcholine, choline, betaine, trimethylamine, and TMAO levels were upgraded. In our study, *Fusobacterium*, *Collinsella*, and *Akkermansia* were responsible for the production of these choline metabolites, especially TMAO. Indeed, manipulating the gut–liver axis may improve the pathological state of various diseases (Milosevic et al., 2019; Jones and Neish, 2021). In the present study, we found that administration and intervention significantly activated hepatic FXR levels, leading to elevated TBA levels. Bile acids are known to play an important role in metabolic diseases (Thomas et al., 2008). They mainly exist in the enterohepatic circulation system and produce protective effect through recycling (Ticho et al., 2019). Thus, our findings demonstrated the importance of gut microbiota in the process of disease, too.

## Conclusion

Taken as a whole, salidroside, tyrosol, and hydroxytyrosol intervention for 84 days could ameliorate the features of MetS in female mice fed with HFru diets, as determined by evaluating serum and liver parameters and analyzing the differential metabolite relationship between liver and intestinal bacteria. The results showed that salidroside, tyrosol, and hydroxytyrosol groups could be able to reduce the levels of LDL-C, TG, GLU, and UA in serum as well as XOD, FMO3, and TNF-α activities in the liver. These beneficial effects may be achieved by regulating some metabolites through the gut–liver axis, such as TMAO, bile acid, and amino acid. In addition, gut microbial community analysis showed that supplementation with salidroside, tyrosol, and hydroxytyrosol reduced *Proteobacteria* abundance and increased *Actinobacteria* abundance, and hydroxytyrosol regulated more effectively than salidroside and tyrosol in the mouse gut. This study provides evidence to support the potential use of salidroside, tyrosol, and hydroxytyrosol to prevent MetS and promote these to process into beneficial dietary components in the future.

## Data Availability

The datasets presented in this study can be found in online repositories. The names of the repository/repositories and accession number(s) can be found below: https://www.ncbi.nlm.nih.gov/geo/, GSE194127.
