# Supplementary material for: Natural Phenylethanoid Supplementation Alleviates Metabolic Syndrome in Female Mice Induced by High-Fructose Diet
Source: Front Pharmacol. 2022 Jul 19;13:850777. doi: 10.3389/fphar.2022.850777 (PMC9343882; doi:10.3389/fphar.2022.850777)
Supplement: Supplementary file 1 [file Table1.DOCX]

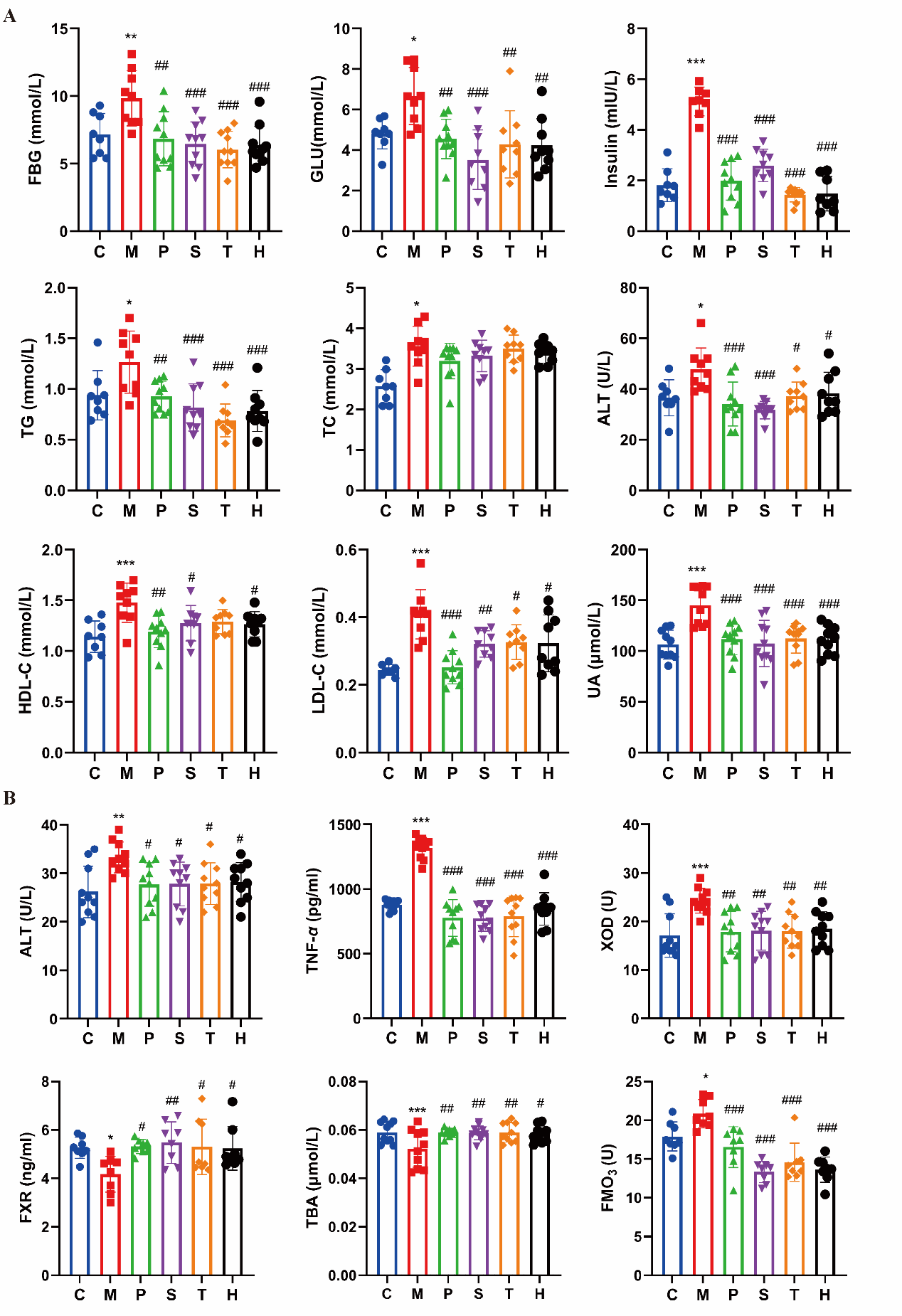


**Figure 1** Effect on fasting blood glucose, serum biochemical indicators (A) and hepatic biochemical indicators (B) in mice. * *P* < 0.05, ** *P* < 0.01, *** *P* < 0.001, *vs* C; ^#^ *P* < 0.05, ^##^ *P* < 0.01, ^###^ *P* < 0.001 *vs* M. C: control; M: model; P: pioglitazone; S: salidroside; T: tyrosol; H: hydroxytyrosol. FBG: fasting blood glucose; ALT: alanine aminotransferase; TC: total cholesterol; TG: triglyceride; HDL-C: high density lipoprotein cholesterol; LDL-C: low density lipoprotein cholesterol; GLU: glucose ; UA: uric acid; INS: insulin; XOD: xanthine oxidase; TNF-α: tumor necrosis factor-α; TBA: total bile acid; FXR: farnesoid X receptor; FMO3: flavin monooxygenase 3.





**Figure 2** Metabolites were identified in the NMR spectrum. (A) The original spectra. (B), (C) and (D) Magnified spectrogram.


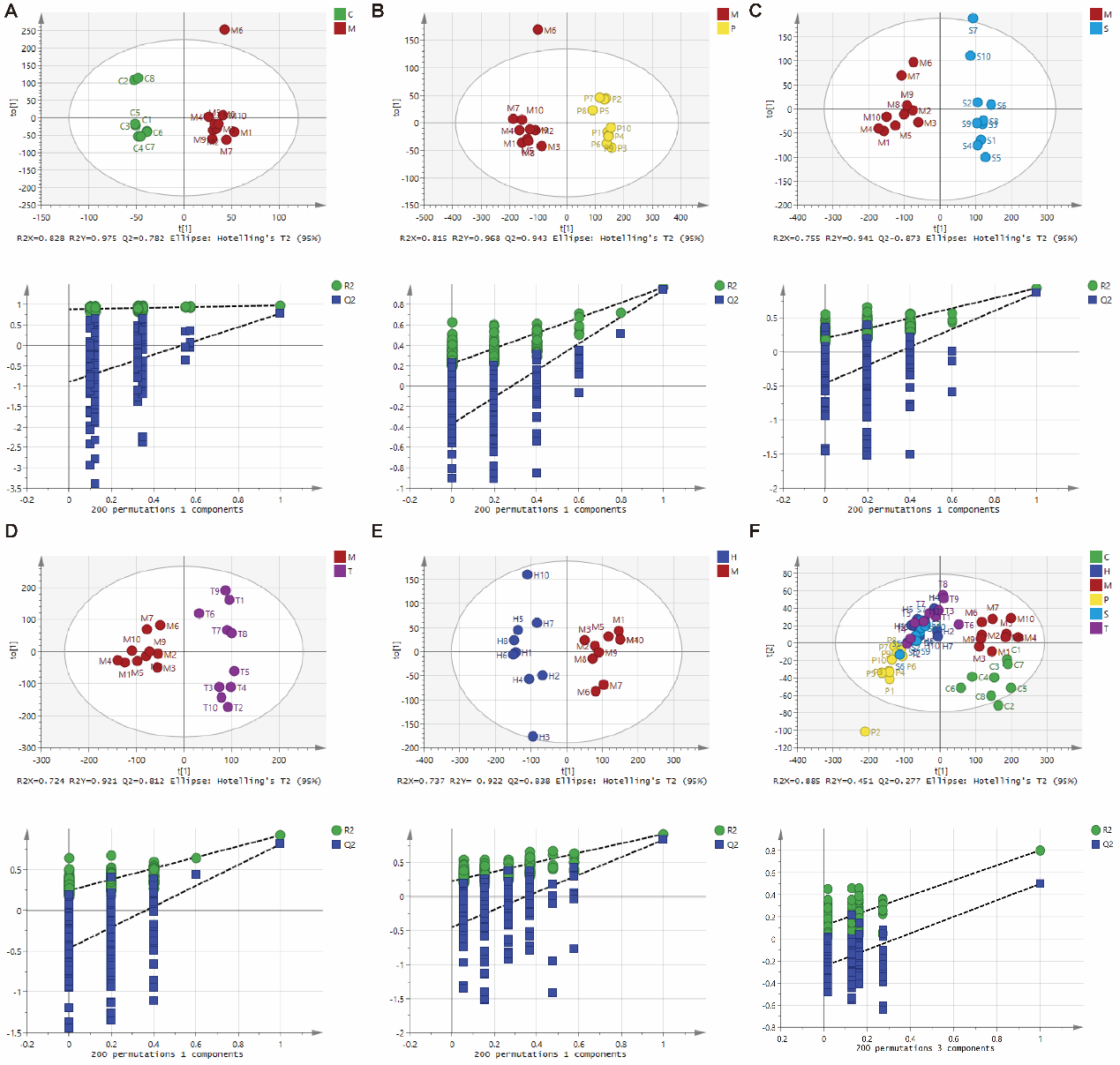


**Figure 3** Score scatter plot of PLS-DA model and Permutation test of OPLS-DA model. (A) C vs M. (B) P vs M. (C) S vs M. (D) T vs M. (E) H vs M. (F) all groups.

The X axis t [1] P denotes the predicted principal component score of the first principal component, the Y axis t [1] O denotes the orthogonal principal component scores in the PLS-DA model. The X axis represents the replacement retention, the Y axis represents the value of R^2^Y or Q^2^, the green dot represents the value of R^2^Y and the blue square represents the value of Q of the replacement test. The dotted lines represent the regression lines of R Y and Q, respectively. C: control; M: model; P: pioglitazone; S: salidroside; T: tyrosol; H: hydroxytyrosol.


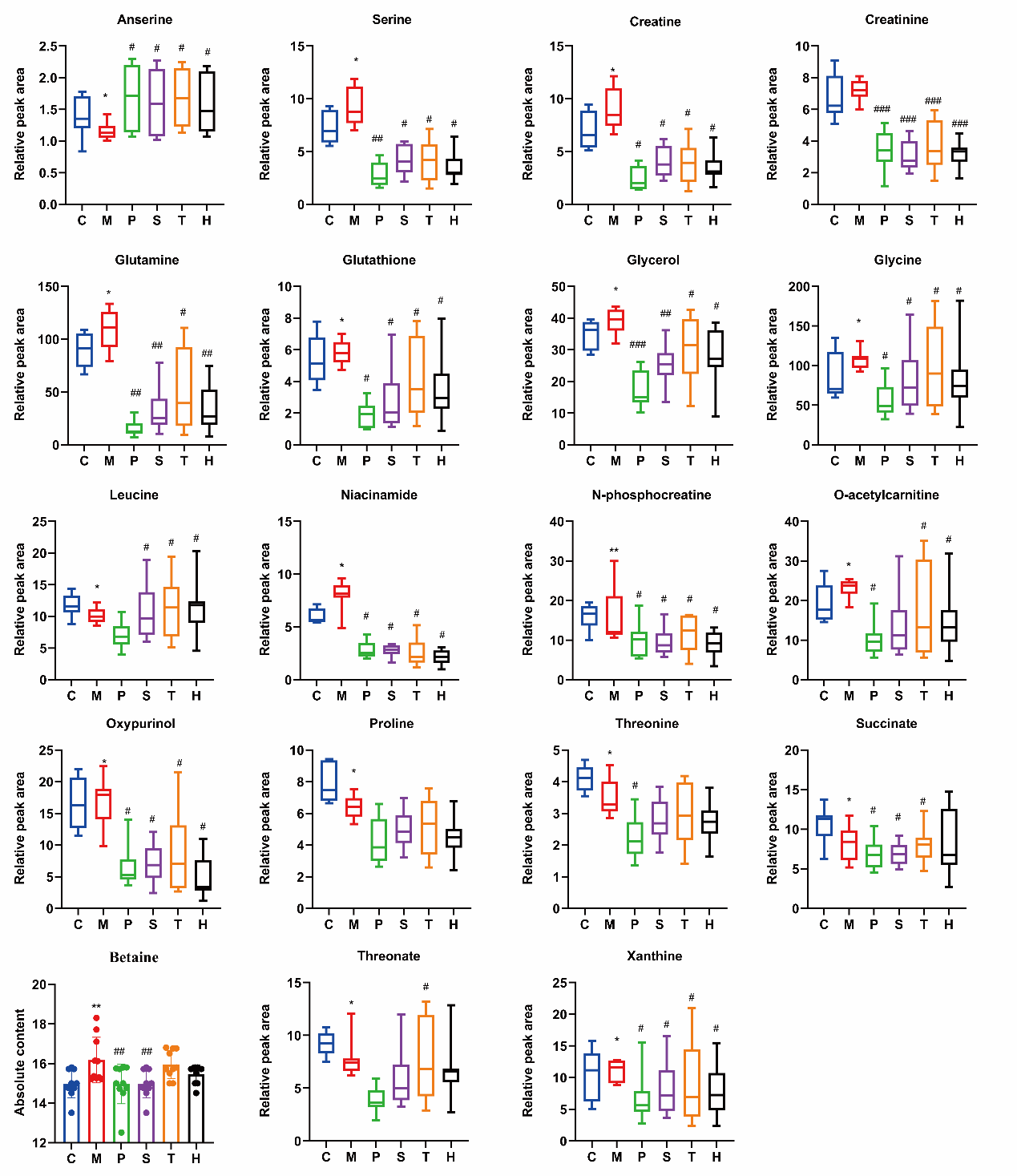


**Figure 4** Other differential metabolites changes in common by boxplot. * *P* < 0.05, ** *P* < 0.01, *** *P* < 0.001, *vs* C; ^#^ *P* < 0.05, ^##^ *P* < 0.01, ^###^ *P* < 0.001 *vs* M. C: control; M: model; P: pioglitazone; S: salidroside; T: tyrosol; H: hydroxytyrosol.


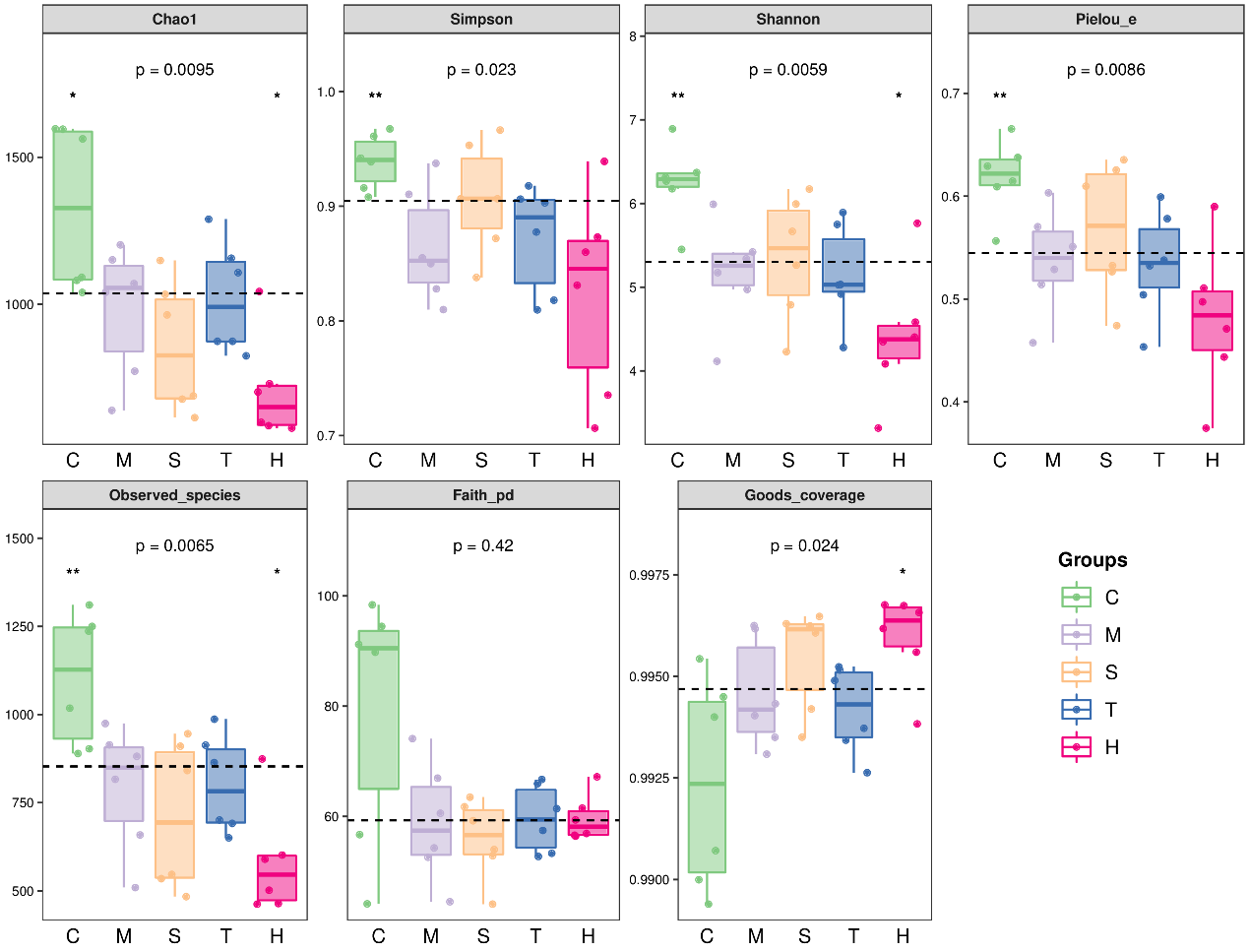


**Figure 5** Alpha diversity index in five groups. C: control; M: model; S: salidroside; T: tyrosol; H: hydroxytyrosol.


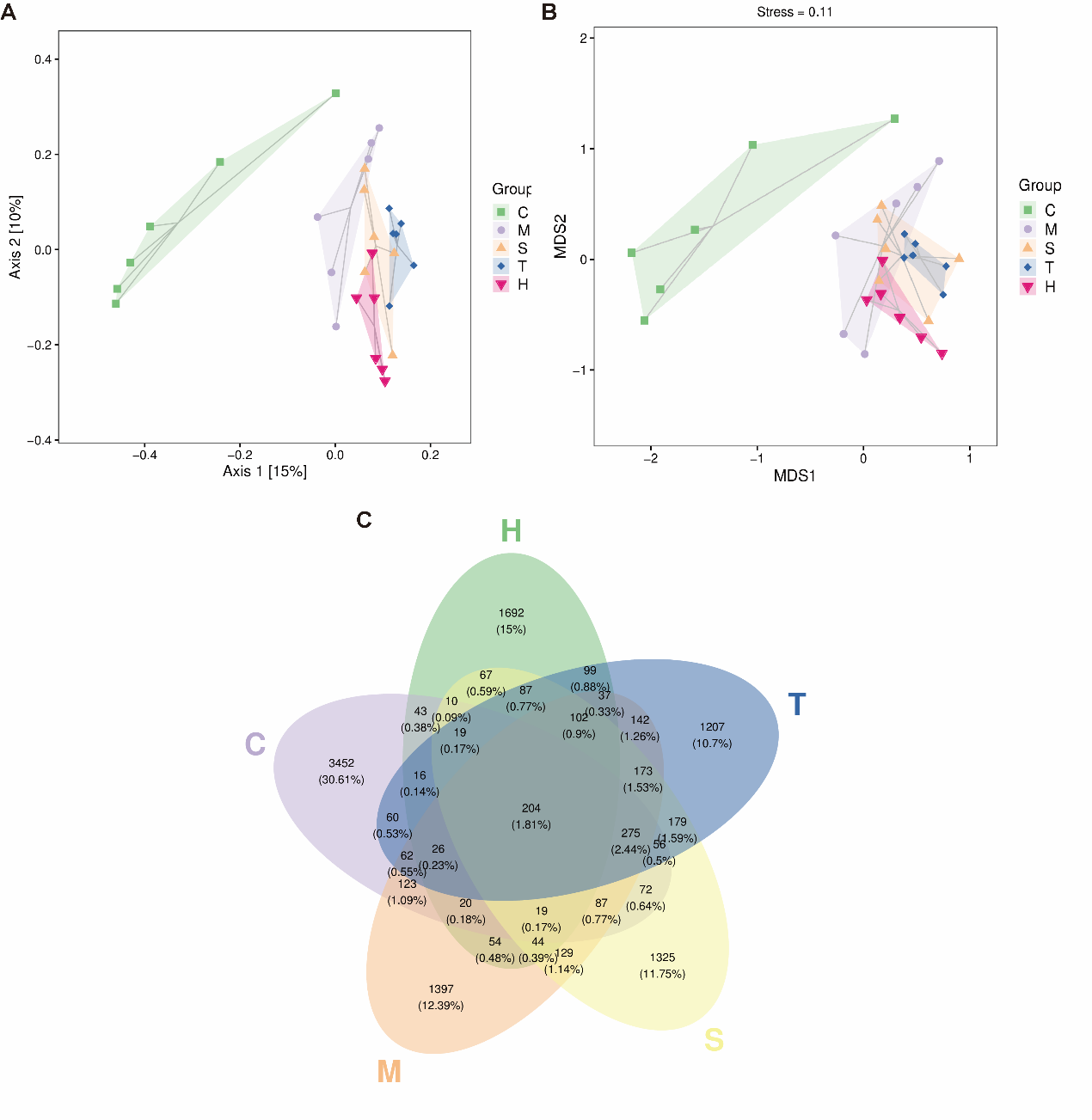


**Figure 6** Salidroside, tyrosol or hydroxytyrosol modulates the composition of gut microbiota. (A) Principal coordinate analysis. (B) Non-metric multidimensional scaling analysis. (C) Venn diagram. C: control; M: model; S: salidroside; T: tyrosol; H: hydroxytyrosol.


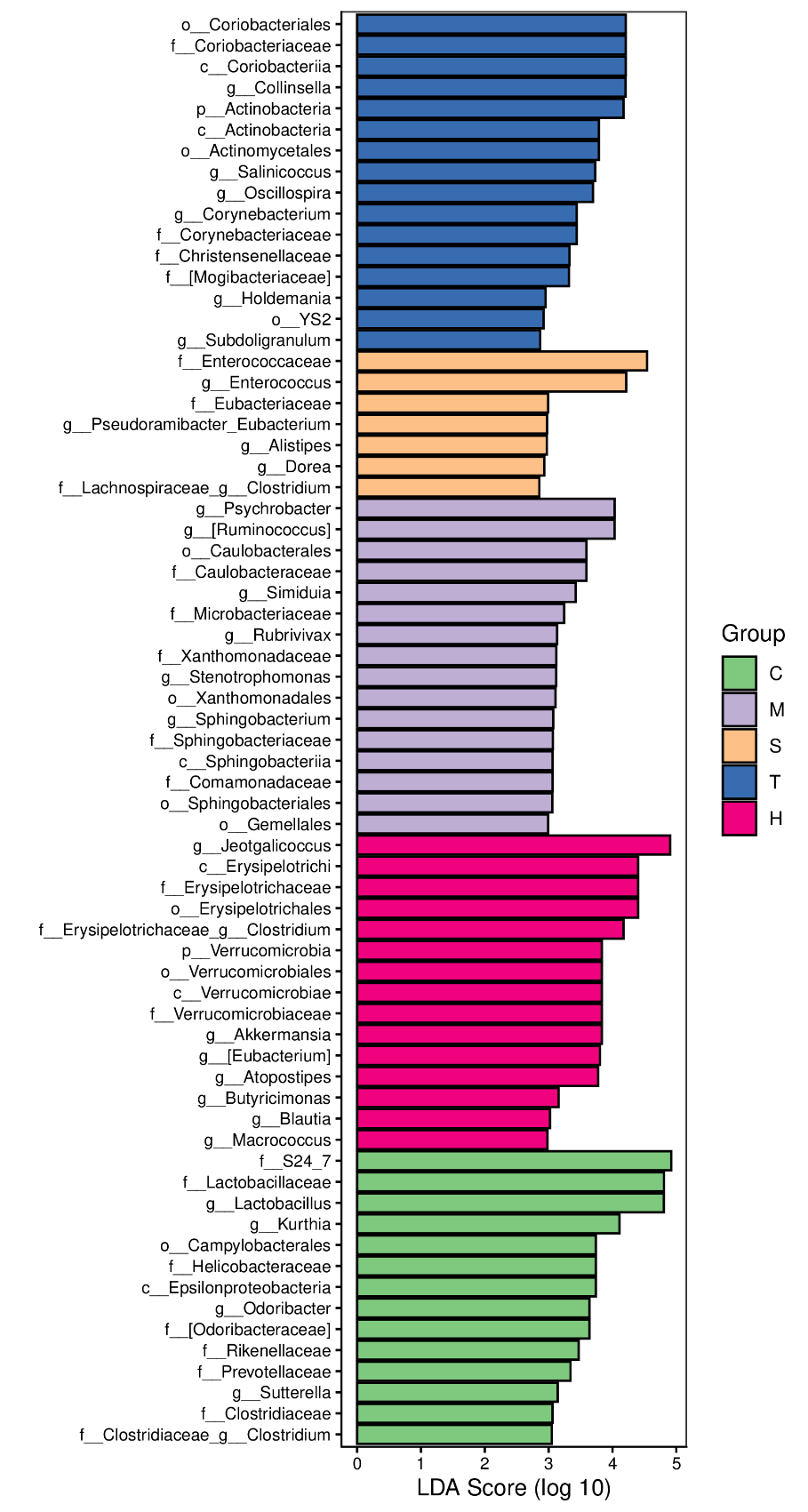


**Figure 7** The threshold of the logarithmic score of LDA analysis was 4.0. C: control; M: model; S: salidroside; T: tyrosol; H: hydroxytyrosol.


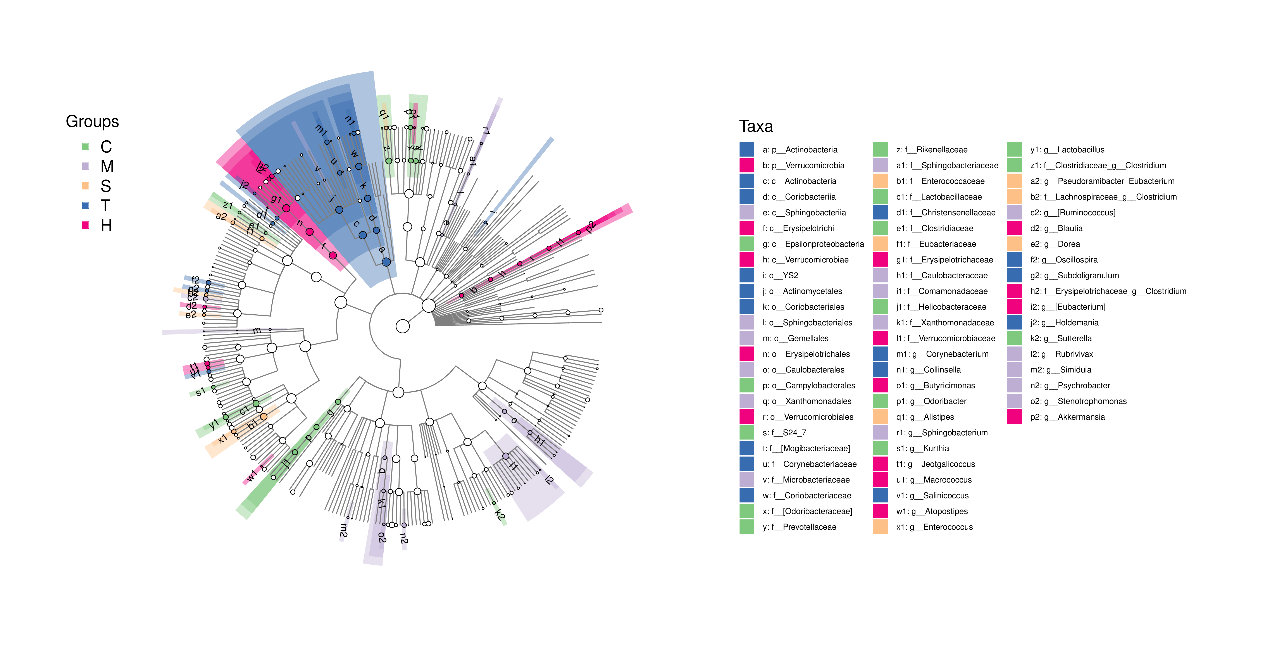


**Figure 8** LEfSe analysis of the dominant biomarker taxaive among the five groups. C: control; M: model; P: pioglitazone; S: salidroside; T: tyrosol; H: hydroxytyrosol.


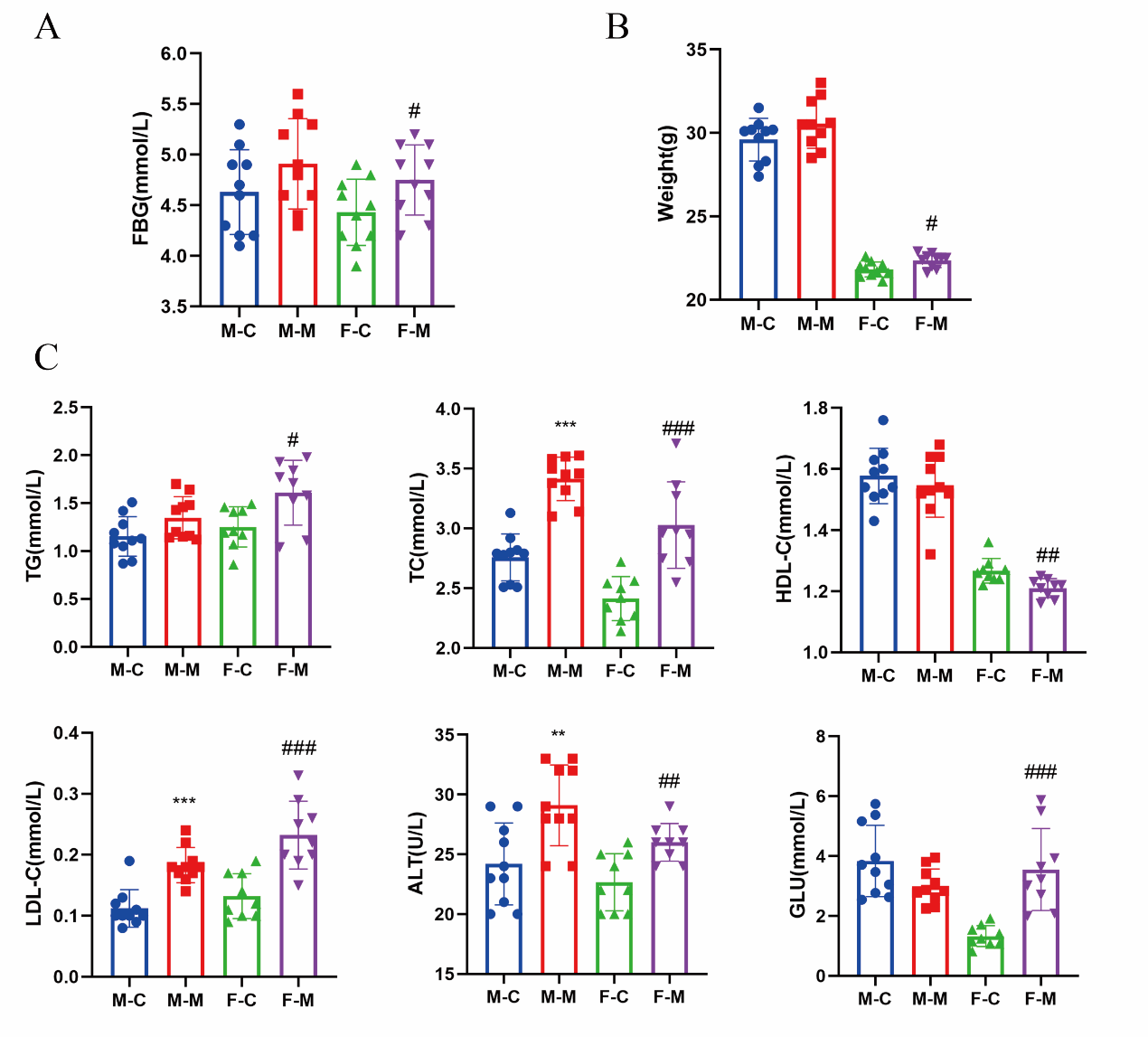


**Figure 9** Fasting blood glucose (A), finial weight (B) and serum TG, TC, HDL-C, LDL-C, ALT, GLU (C) were measured. * *P* < 0.05, ** *P* < 0.01, *** *P* < 0.001, M-M vs M-C; ^#^ *P* < 0.05, ^##^ *P* < 0.01, ^###^ *P* < 0.001 F-M vs F-C. M-C: male mice male mice; M-M: male mice model; F-C: female mice male mice; F-M: female mice mode; M-C: tyrosol. FBG: fasting blood glucose; ALT: alanine aminotransferase; TC: total cholesterol; TG: triglyceride; HDL-C: high density lipoprotein cholesterol; LDL-C: low density lipoprotein cholesterol; GLU: glucose.

**Table 1** Specific information on metabolites identified by NMR

| 编号 | 代谢物 | 化学位移 | 基团 |
| --- | --- | --- | --- |
| 1 | Isoleucine | 0.94 (d),1.02 (d),1.26 (m),  1.47 (m),1.98 (m),3.67 (d) | *δ*-CH_3_, *γ'*-CH_3_, *γ*-CH_2_,  *β*-CH, *α*-CH |
| 2 | Leucine | 0.97 (t),1.73 (ddd),3.74 (m) | δ-CH3, β-CH2, γ-CH, α-CH |
| 3 | Valine | 1.00 (d),1.05 (d),2.27 (m),  3.60 (d) | *γ′*-CH_3_, *γ-*CH_3_, *β*-CH, *α*-CH |
| 4 | 3-hydroxybutyrate | 1.21 (d),2.27 (dd),2.40 (dd),  4.15(q) | CH_3_, CH_2_, CH |
| 5 | Lactate | 1.33 (d), 4.12 (q) | CH_3_, CH |
| 6 | Threonine | 1.32 (d), 4.24 (m) | *γ*-CH_3_, *β*-CH |
| 7 | Alanine | 1.49 (d), 3.79 (t) | *β*-CH_3_, *α*-CH |
| 8 | Acetate | 1.93 (s) | CH_3_ |
| 9 | Proline | 2.06 (m) | *β*-CH_2_ |
| 10 | Glutamate | 2.43 (m),3.72 (m) | *γ*-CH_3_, *α*-CH |
| 11 | *O*-acetylcarnitine | 2.13 (s), 2.61 (dd),  5.57 (q) | O=C-CH_3_, CH_2_,  CH |
| 12 | Succinate | 2.42 (s) | CH_2_ |
| 13 | Malate | 2.66 (m), 4.46 (d) | CH_2_, CH |
| 14 | Trimethylamine | 2.94 (s) | CH_3_ |
| 15 | Glutathione | 2.97 (dd), 3.78 (m) | CH_2_, CH_2_ |
| 16 | Glutamine | 2.15 (m),2.35 (m),3.75 (dd) | 7-CH_2_, 4-CH |
| 17 | Cadaverine | 3.02 (t) | 6-CH_2_, 4-CH_2_ |
| 18 | Creatine | 3.05 (s), 3.91 (s) | CH_3_, CH_2_ |
| 19 | Creatinine | 3.03 (s), 4.05 (s) | -*N*(CH_3_), 3-CH_2_ |
| 20 | Choline | 3.21 (s), 4.07 (td) | -*N*(CH_3_)_3_ |
| 21 | Phosphorylcholine | 3.21 (s) | -*N*(CH_3_)_3_ |
| 22 | Carnitine | 3.22 (s) | -*N*(CH_3_)_3_ |
| 23 | GPC | 3.27 (s) | -*N*(CH_3_)_3_ |
| 24 | Taurine | 3.32 (t), 3.40 (t) | *N*-CH_2_, S-CH_2_ |
| 25 | TMAO | 3.29 (s) | -*N*(CH_3_)_3_ |
| 26 | Methanol | 3.36 (s) | CH_3_ |
| 27 | Glycerol | 3.55 (m), 3.64 (m) | CH_2_, CH |
| 28 | Glycine | 3.57 (s) | CH_2_, CH |
| 29 | *N*-phosphocreatine | 3.93 (s) | CH_2_ |
| 30 | Threonate | 4.01 (m) | 3-CH2, 2-CH, 5-CH |
| 31 | Uridine | 5.90 (t),7.87 (t) | 5-CH, 6-CH |
| 32 | Inosine | 8.19 (s), 8.31 (s) | 2-CH, 7-CH, |
| 33 | Glucose | 4.66 (d), 5.25 (d) | (*β*) 1-CH |
| 34 | Serine | 3.96 (m) | 2-CH_2_ |
| 35 | Guanosine | 4.75 (d) | 3-CH |
| 36 | Mannose | 5.17 (d) | 1-CH |
| 37 | Glycogen | 5.40 (s) | 1-CH |
| 38 | Glucose-1-phosphate | 5.46 (m) | 1-CH |
| 39 | Uracil | 5.81 (d), | 5-CH, 6-CH |
| 40 | ADP + ATP | 6.11 (d), 8.25 (s) | 2-CH, 12-CH, 7-CH |
| 41 | Fumarate | 6.53 (s) | CH |
| 42 | Anserine | 7.12 (d) | 8-CH |
| 43 | Tyramine | 6.92 (d), 7.20 (d) | 2-CH, 6-CH,  3-CH, 5-CH |
| 44 | Phenylalanine | 7.32 (d), 7.36 (m) | 2-CH, 6-CH |
| 45 | Thymine | 7.36 (d), 1.93 (s) | 5-CH_3_, 6-CH |
| 46 | Xanthosine | 7.86 (s) | 7-CH |
| 47 | Xanthine | 7.94 (s) | 7-CH |
| 48 | Oxypurinol | 8.30 (s) | 9-CH |
| 49 | Hypoxanthine | 8.15 (s), 8.21 (s) | 2-CH, 7-CH |
| 50 | Adenosine | 8.39 (s), 8.26 (s), | 2-CH, 8-CH, |
| 51 | Formate | 8.48 (s) | CHO |
| 52 | Niacinamide | 5.62 (dd), 8.73 (dd) | 5-CH, 6-CH |

**Table 2** OPLS-DA and 200 permutation test parameter

| Group | R^2^X | R^2^Y | Q^2^ | R^2^ intercepts | Q^2^ intercepts |
| --- | --- | --- | --- | --- | --- |
| C *vs* M | 0.828 | 0.975 | 0.782 | 0.883 | -0.834 |
| P *vs* M | 0.815 | 0.968 | 0.943 | 0.231 | -0.333 |
| S *vs* M | 0.755 | 0.941 | 0.873 | 0.184 | -0.491 |
| T *vs* M | 0.724 | 0.921 | 0.812 | 0.245 | -0.465 |
| H *vs* M | 0.737 | 0.922 | 0.838 | 0.243 | -0.429 |
| All | 0.885 | 0.451 | 0.277 | 0.118 | -0.282 |

**Table 3** Differential metabolites in common between control and model group (differential screening criterion: VIP > 1 and p-value < 0.05).

| metabolites | ppm | p-value | VIP | Fold change |
| --- | --- | --- | --- | --- |
| 3-Hydroxybutyrate | 1.21 | 1.20E-03 | 1.33 | 1.7 |
| Leucine | 0.97 | 3.88E-02 | 1.05 | 1.2 |
| Lactate | 1.34 | 3.88E-02 | 1.38 | 1.2 |
| Threonine | 4.26 | 2.59E-02 | 1.24 | 1.2 |
| Alanine | 3.78 | 4.47E-02 | 1.97 | 0.9 |
| Proline | 4.16 | 4.90E-03 | 1.14 | 1.2 |
| *O*-acetylcarnitine | 2.15 | 4.80E-02 | 1.21 | 1.1 |
| Succinate | 2.41 | 4.28E-02 | 1.24 | 1.3 |
| Trimethylamine | 2.94 | 1.90E-03 | 2.31 | 2.5 |
| Glutathione | 2.95 | 1.70E-03 | 1.09 | 1.2 |
| Glutamine | 3.75 | 3.23E-02 | 3.84 | 0.8 |
| Creatine | 3.94 | 3.75E-02 | 1.25 | 0.8 |
| Creatinine | 4.04 | 3.91E-02 | 1.02 | 1.2 |
| Choline | 3.22 | 1.43E-02 | 1.16 | 1.4 |
| Phosphorylcholine | 3.23 | 3.81E-02 | 6.24 | 2.4 |
| GPC | 3.27 | 1.69E-02 | 4.99 | 2.1 |
| Taurine | 3.32 | 5.90E-03 | 7.96 | 2.7 |
| TMAO | 3.29 | 2.23E-02 | 4.70 | 1.5 |
| Glycerol | 3.56 | 3.83E-02 | 1.73 | 0.9 |
| Glycine | 3.58 | 1.20E-03 | 5.83 | 1.3 |
| *N*-phosphocreatine | 3.92 | 7.30E-03 | 3.83 | 2.5 |
| Threonate | 4.02 | 3.91E-02 | 1.02 | 1.2 |
| Inosine | 4.29 | 2.00E-03 | 1.08 | 1.2 |
| Glucose | 4.68 | 2.90E-02 | 2.03 | 0.8 |
| Serine | 3.96 | 2.91E-02 | 1.23 | 0.8 |
| Anserine | 6.83 | 4.29E-09 | 1.25 | 17.3 |
| Xanthine | 7.94 | 3.10E-03 | 1.63 | 1.3 |
| Oxypurinol | 8.30 | 4.48E-02 | 1.43 | 1.2 |
| Niacinamide | 8.29 | 1.00E-03 | 1.27 | 0.7 |

**Table 4** Differential metabolites in common between pioglitazone and model group (differential screening criterion: VIP > 1 and p-value < 0.05).

| metabolites | ppm | p-value | VIP | Fold change |
| --- | --- | --- | --- | --- |
| Leucine | 0.97 | 7.00E-04 | 1.05 | 0.7 |
| Valine | 1.05 | 1.04E-07 | 1.14 | 0.4 |
| 3-Hydroxybutyrate | 1.21 | 2.00E-04 | 1.03 | 2.1 |
| Lactate | 1.34 | 1.51E-10 | 3.72 | 0.2 |
| Alanine | 1.50 | 2.06E-08 | 3.09 | 0.3 |
| Acetate | 1.93 | 3.25E-02 | 1.30 | 0.7 |
| *O*-acetylcarnitine | 2.15 | 4.01E-08 | 1.55 | 0.4 |
| Succinate | 2.41 | 1.00E-04 | 2.05 | 0.3 |
| Trimethylamine | 2.94 | 1.70E-03 | 1.16 | 0.6 |
| Glutathione | 2.95 | 1.71E-09 | 1.09 | 0.3 |
| Isoleucine | 3.67 | 1.84E-05 | 2.00 | 0.3 |
| Creatine | 3.94 | 2.28E-02 | 1.44 | 0.4 |
| Choline | 3.22 | 4.75E-02 | 1.39 | 1.6 |
| Phosphorylcholine | 3.23 | 1.50E-03 | 1.46 | 0.5 |
| Carnitine | 3.24 | 4.71E-09 | 2.29 | 0.3 |
| GPC | 3.27 | 2.44E-11 | 5.67 | 0.1 |
| Taurine | 3.32 | 1.66E-05 | 3.43 | 0.6 |
| TMAO | 3.29 | 3.48E-10 | 6.38 | 0.2 |
| Glycerol | 3.56 | 4.73E-12 | 4.16 | 0.2 |
| Glycine | 3.58 | 1.52E-12 | 4.61 | 0.2 |
| *N*-phosphocreatine | 3.92 | 5.69E-05 | 1.09 | 0.6 |
| Threonate | 4.02 | 1.20E-05 | 1.02 | 0.5 |
| Inosine | 4.29 | 1.92E-08 | 1.02 | 0.3 |
| Glucose | 5.28 | 1.40E-12 | 5.67 | 0.1 |
| Glucose-1Phosphate | 5.45 | 1.60E-03 | 2.02 | 0.1 |
| Oxypurinol | 8.30 | 1.53E-06 | 1.35 | 0.4 |

**Table 5** Differential metabolites in common between salidroside and model group (differential screening criterion: VIP > 1 and p-value < 0.05).

| metabolites | ppm | p-value | VIP | Fold change |
| --- | --- | --- | --- | --- |
| Leucine | 0.97 | 3.02E-02 | 1.05 | 0.8 |
| Valine | 1.05 | 9.70E-03 | 1.07987 | 0.6 |
| 3-Hydroxybutyrate | 1.21 | 4.91E-05 | 1.03499 | 2.1 |
| Lactate | 1.34 | 4.55E-06 | 9.1998 | 0.4 |
| Alanine | 1.50 | 1.01E-06 | 3.4204 | 0.3 |
| Acetate | 1.93 | 2.61E-02 | 1.14284 | 0.7 |
| Succinate | 2.41 | 2.21E-04 | 2.44144 | 0.3 |
| Trimethylamine | 2.94 | 1.10E-05 | 1.01337 | 0.5 |
| Isoleucine | 3.67 | 1.11E-03 | 2.06442 | 0.4 |
| Creatine | 3.94 | 1.11E-02 | 1.43708 | 0.4 |
| Creatinine | 4.04 | 9.73E-10 | 1.00699 | 0.4 |
| Choline | 3.22 | 2.52E-02 | 1.46077 | 0.9 |
| Phosphorylcholine | 3.23 | 5.78E-03 | 1.64824 | 0.5 |
| Carnitine | 3.24 | 5.15E-07 | 2.53721 | 0.4 |
| GPC | 3.27 | 2.95E-07 | 5.93414 | 0.3 |
| Taurine | 3.32 | 1.86E-04 | 4.15104 | 0.6 |
| TMAO | 3.29 | 7.86E-06 | 6.25229 | 0.4 |
| Glycerol | 3.56 | 1.88E-07 | 4.31977 | 0.3 |
| Glycine | 3.58 | 6.30E-07 | 4.65643 | 0.4 |
| *N*-phosphocreatine | 3.92 | 3.57E-02 | 1.08698 | 0.7 |
| Inosine | 4.29 | 2.13E-06 | 1.09462 | 0.4 |
| Glucose | 5.28 | 9.95E-07 | 5.78075 | 0.3 |
| Serine | 3.96 | 3.97E-03 | 1.80021 | 0.3 |
| Glucose-1-phosphate | 5.45 | 3.93E-02 | 1.85994 | 0.3 |
| Fumarate | 6.53 | 3.47E-05 | 1.01767 | 0.3 |
| Oxypurinol | 8.30 | 5.18E-06 | 1.54698 | 0.4 |

**Table 6** Differential metabolites in common between tyrosol and model group (differential screening criterion: VIP > 1 and p-value < 0.05).

| metabolites | ppm | p-value | VIP | Fold change |
| --- | --- | --- | --- | --- |
| 3-Hydroxybutyrate | 1.21 | 3.71E-04 | 1.15266 | 1.8 |
| Lactate | 1.34 | 1.17E-04 | 9.99113 | 0.3 |
| Threonine | 4.26 | 1.00E-04 | 1.24 | 0.8 |
| Alanine | 1.50 | 2.31E-04 | 3.66636 | 0.4 |
| Acetate | 1.93 | 2.79E-02 | 1.52119 | 0.6 |
| *O*-acetylcarnitine | 2.15 | 9.43E-03 | 1.02267 | 0.5 |
| Succinate | 2.41 | 1.66E-03 | 2.66336 | 0.4 |
| Trimethylamine | 2.94 | 7.74E-03 | 1.01337 | 0.5 |
| Glutathione | 4.04 | 3.88E-07 | 1.01229 | 0.4 |
| Choline | 3.22 | 4.44E-03 | 1.43551 | 0.8 |
| Phosphorylcholine | 3.23 | 5.40E-06 | 1.42317 | 0.6 |
| Carnitine | 3.24 | 1.64E-06 | 2.82375 | 0.4 |
| GPC | 3.27 | 1.03E-03 | 6.0084 | 0.4 |
| Taurine | 3.32 | 8.55E-03 | 4.40552 | 0.5 |
| TMAO | 3.29 | 5.21E-03 | 6.1719 | 0.6 |
| Glycine | 3.58 | 1.46E-03 | 4.682 | 0.4 |
| *N*-phosphocreatine | 3.92 | 3.73E-02 | 1.17662 | 0.8 |
| Inosine | 4.29 | 1.62E-04 | 1.20917 | 0.3 |
| Glucose | 5.28 | 2.90E-03 | 5.65135 | 0.4 |

**Table 7** Differential metabolites in common between hydroxytyrosol and model group (differential screening criterion: VIP > 1 and p-value < 0.05).

| metabolites | ppm | p-value | VIP | Fold change |
| --- | --- | --- | --- | --- |
| 3-Hydroxybutyrate | 1.21 | 3.71E-04 | 1.15266 | 1.8 |
| Lactate | 1.34 | 1.17E-04 | 9.99113 | 0.3 |
| Threonine | 4.26 | 1.00E-04 | 1.24 | 0.8 |
| Alanine | 1.50 | 2.31E-04 | 3.66636 | 0.4 |
| Acetate | 1.93 | 2.79E-02 | 1.52119 | 0.6 |
| *O*-acetylcarnitine | 2.15 | 9.43E-03 | 1.02267 | 0.5 |
| Succinate | 2.41 | 1.66E-03 | 2.66336 | 0.4 |
| Trimethylamine | 2.94 | 7.74E-03 | 1.01337 | 0.5 |
| Glutathione | 4.04 | 3.88E-07 | 1.01229 | 0.4 |
| Choline | 3.22 | 4.44E-03 | 1.43551 | 0.8 |
| Phosphorylcholine | 3.23 | 5.40E-06 | 1.42317 | 0.6 |
| Carnitine | 3.24 | 1.64E-06 | 2.82375 | 0.4 |
| GPC | 3.27 | 1.03E-03 | 6.0084 | 0.4 |
| Taurine | 3.32 | 8.55E-03 | 4.40552 | 0.5 |
| TMAO | 3.29 | 5.21E-03 | 6.1719 | 0.6 |
| Glycine | 3.58 | 1.46E-03 | 4.682 | 0.4 |
| *N*-phosphocreatine | 3.92 | 3.73E-02 | 1.17662 | 0.8 |
| Inosine | 4.29 | 1.62E-04 | 1.20917 | 0.3 |
| Glucose | 5.28 | 2.90E-03 | 5.65135 | 0.4 |
